# The mismatch between the health research and development (R&D) that is needed and the R&D that is undertaken: an overview of the problem, the causes, and solutions

**DOI:** 10.3402/gha.v6i0.22450

**Published:** 2013-10-10

**Authors:** Roderik F. Viergever

**Affiliations:** 1 Department of Primary and Community Care, Radboud University Nijmegen Medical Center, Nijmegen, The Netherlands; 2 Faculty of Public Health and Policy, London School of Hygiene and Tropical Medicine, London, UK

**Keywords:** R&D, priorities, agenda, pharmaceutical industry, research funding

## Abstract

One of the most pressing global health problems is that there is a mismatch between the health research and development (R&D) that is needed and that which is undertaken. The dependence of health R&D on market incentives in the for-profit private sector and the lack of coordination by public and philanthropic funders on global R&D priorities have resulted in a global health R&D landscape that neglects certain products and populations and is characterised, more generally, by a distribution that is not ‘needs-driven’. This article provides an overview of the mismatch, its causes, and solutions.

The mismatch between the health research and development (R&D)^1^ that is needed and that which is undertaken was first demonstrated in 1990, when it was shown that less than 10% of global health research expenditure was spent on the health problems of developing countries, which then represented more than 90% of the world's burden of preventable mortality (Fig. 1) (1–4). This disparity later became well known as the ‘10/90-gap’. The nature of the 10/90-gap has changed substantially since 1990: the distribution of the global disease burden has changed (5); overall global funding for health R&D has increased from 30 billion USD in 1986 to 240 billion USD in 2010 (6); there are many more and new types of actors involved in health R&D (7–9); and a variety of new approaches to innovation have been suggested and tested in recent years, and continue to be developed, to encourage action on previously neglected areas of health R&D (10). However, even though the nature of the 10/90-gap has changed since 1990, the gap itself very much remains to this day (6).

**Fig. 1:**
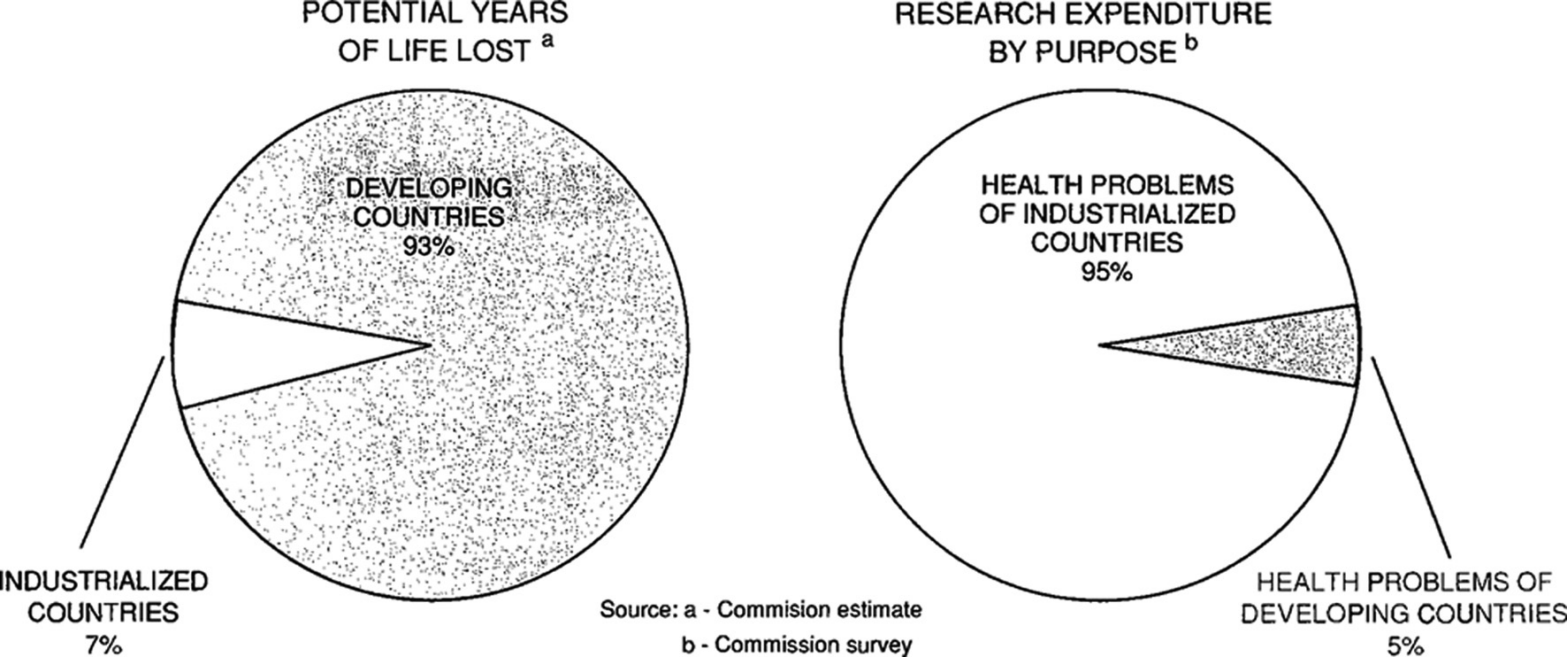
The figure from the report of the Commission on Health Research for Development that formed the basis for the term ‘10/90-gap’ (reprinted by permission of Oxford University Press, USA) (4).

The 10/90-gap is a prominent expression of a broader problem which is better described as one of ‘neglected populations’ (10). This neglect can be seen in the lack of R&D for diseases that predominantly affect developing countries (the ‘neglected diseases’) (11, 12), in the lack of R&D that addresses the specific needs of developing countries in relation to diseases with a global incidence, and in the lack of development of affordable medicines for all (10). But the problem of neglect extends beyond the developing world, as becomes clear from the global lack of R&D for new antibiotics (13), appropriate children's medicines (and other products) (14, 15), and orphan diseases (7, 16). In addition to neglected populations, there are neglected products. R&D is generally more focused on the development of drugs and vaccines than on the development of diagnostics or platform technologies (technologies that can potentially be applied to different diseases and products) (11, 17). Moreover, for specific diseases, some products are neglected in terms of R&D, whereas others are not (18).

Besides the discrete distinction between neglected and non-neglected areas of health R&D, there is a broader issue with the global distribution of health R&D as part of the mismatch. ‘Needs-driven’ R&D is not necessarily characterised by a linear relationship between disease burden and R&D funding, because burden of disease is just one of the factors that determine health R&D need (see Box 1 for what determines health R&D need) (11, 19). In assessing health R&D needs, it is necessary to be specific about the knowledge and products that are needed for each health problem and to take into account differences in need between different populations (18). However, on the presumption that R&D funding is responsive to the scale of a health problem, a degree of correlation between the burden of a health problem and R&D funding can be expected (11, 19, 20–23). Working from this presumption provides us with a crude approach to assessing the global distribution of health R&D funding (as was done with the 10/90-gap) (4). Within the area of neglected diseases, the Global Funding of Innovation for Neglected Diseases (G-FINDER) reports have shown that of 31 neglected diseases, some are more neglected than others (12). There are three ‘top tier’ diseases which each receive one-third to one-sixth of the total global neglected disease R&D funding, a number of ‘second tier’ diseases which each receive 1–8% of total funding, and several ‘third tier’ diseases, which are the most poorly funded and receive less than 0.5% of the global funding each (12). Table 1 shows the neglected diseases from the most recent G-FINDER report in terms of funding and in terms of global burden of disease. In interpreting this table, it is important to remember that health R&D need depends on more than burden of disease (Box 1). Nonetheless, the findings from G-FINDER make clear the variations in R&D investments for these diseases. Moreover, the G-FINDER reports have shown that R&D investments for a particular disease are not necessarily allocated towards developing the knowledge or products that are most needed for that disease (26). It is concluded that ‘R&D funding is often poorly matched with disease needs and scientific and technical possibilities’ (26).

**Table 1:** Distribution of global health R&D funding across neglected diseases

Disease	Global R&D funding 2011 (million US$)	Global BoD (million DALYs)	Global R&D funding in US$/global DALY
HIV	1,117	81.5	13.7
Malaria	596	82.7	7.2
Tuberculosis	584	49.4	11.8
Dengue	249	0.8	301.7
Diarrhoeal diseases	169	89.5	2.0
Kinetoplastids	142	4.4	32.0
Bacterial pneumonia and meningitis	107	68.0–104.9^a^	1.0–1.6
Helminths (worms and flukes)	90	12.3	7.3
Salmonella infections	48	17.1	2.8
Trachoma	10	0.3	31.1
Leprosy	8	0.006	1400.9
Buruli ulcer	6	NA^b^	–
Rheumatic fever	1	10.1	0.1

*Notes*: Table is based on Table 2 from the G-FINDER report 2012, which reports on global R&D funding for 31 neglected diseases (12). ‘Neglected diseases’ are defined in this report as diseases that disproportionally affect people in developing countries, for which there is a need for new products, and for which there is market failure. The list of 31 diseases includes HIV, tuberculosis and malaria, and thus adheres to a different definition of neglected diseases than WHO (24). Burden of disease (BoD) data are from the Global Burden of Disease (GBD) study 2010 (25). DALYs=disability-adjusted life years.

a Global BoD for bacterial pneumonia and meningitis is displayed as a range because ‘other LRIs’ and ‘other meningitis’ in the GBD study 2010 were not sub-specified into viral or bacterial pathogens. The lower limit represents the BoD without the ‘other’ categories, while the upper limit includes the ‘other categories’.

b Global BoD data for buruli ulcer were not available.

Although there are also indications in other areas, such as R&D for orphan drugs, that there are some diseases that are more neglected than others (16), analyses such as the G-FINDER reports, which aggregate all global funding towards a set of diseases, are rare. Because only few funders publicly report disaggregated statistics on health R&D expenditures, and because of a lack of uniformity in the use of R&D classification systems across different funders, such analyses are complex and resource-intensive (27). However, when we look at individual R&D funders’ investment portfolios, marked variations in funding for similar diseases also become apparent. Brower argued in 2005 that ‘research funding is not necessarily allocated to those who need it most’ by showing the variation in R&D funding for different diseases by the US National Institutes of Health (NIH) (28). Table 2 shows an updated list of US NIH R&D funding for different cancers in the US and makes clear the variation in R&D funding per US disability-adjusted life year (DALY) for these diseases.

**Box 1. What is ‘health R&D need’?**

What determines whether there is a need for health R&D? To determine health R&D need it is necessary to first evaluate which health problems exist that cause a burden of disease (Fig. 2). The more prominent the health problem, the larger the potential impact of R&D. The scale of different health problems is regularly assessed as part of the Global Burden of Disease studies (75, 76). Second, it is necessary to determine the need for new knowledge and/or products (including devices, medicines, vaccines, procedures, and systems (77)) for a given health problem (12, 36–38). Finally, to determine health R&D need, we must also take into account what health R&D is already being undertaken (19, 45, 78–80).

With all these steps it is critical to be specific and account for potential differences in health R&D need between different populations, such as geographical regions, age groups, and socioeconomic sub-groups (11, 18, 81, 82).

**Fig. 2:**
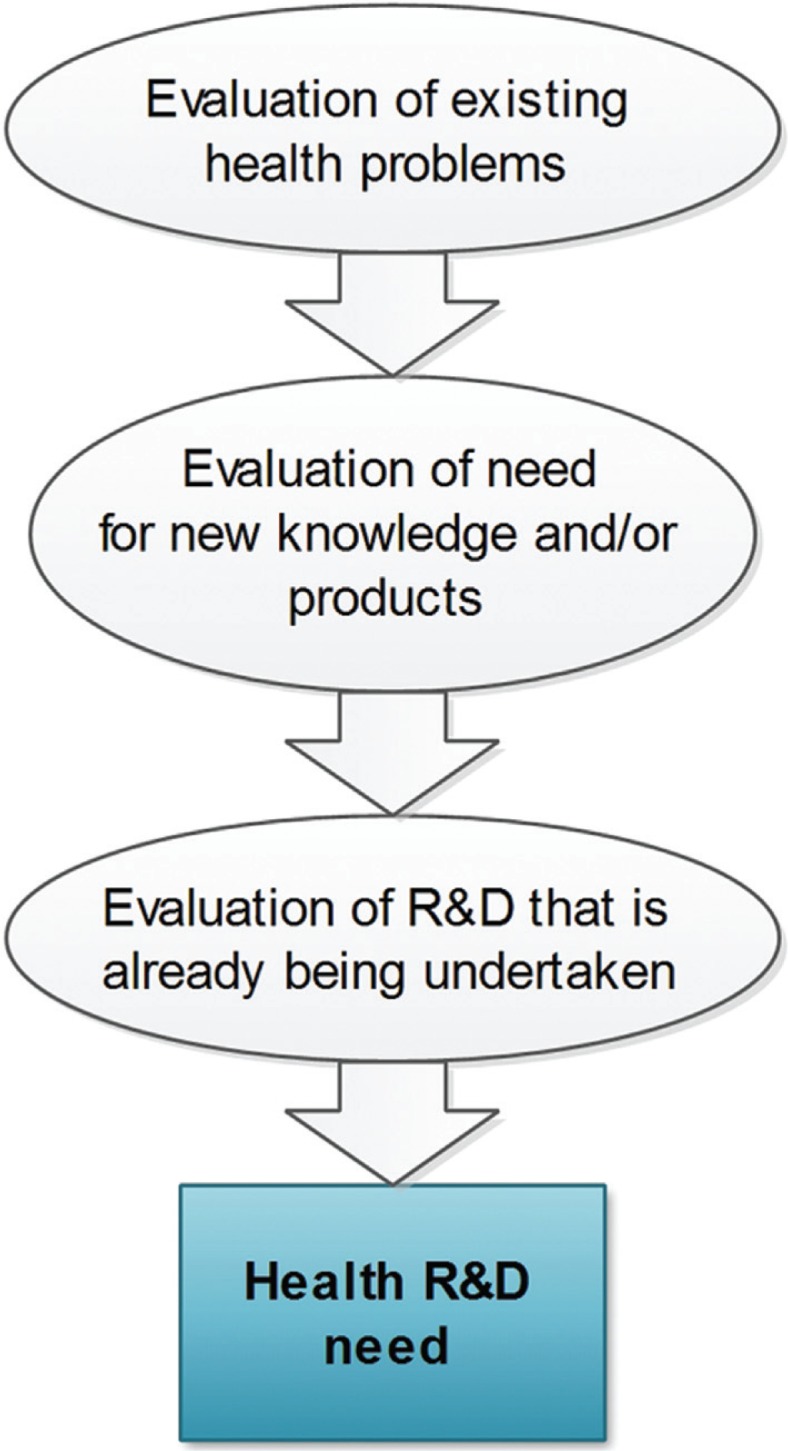
Health R&D need is determined by: (A) existing health problems, (B) the need for new knowledge and products, and (C) the health R&D that is already being undertaken.

**Table 2:** Distribution of US National Institutes of Health (NIH) funding across cancers

Disease	US NIH R&D funding 2011 (million US$)	US BoD (thousand DALYs)	US NIH R&D funding in US$/US DALY
Prostate cancer	284	225	1262.0
Cervical cancer	143	114	1253.7
Breast cancer	715	612	1167.4
Ovarian cancer	138	145	951.8
Colorectal cancer	313	542	577.4
Liver cancer	74	138	537.0
Uterine cancer	40	75	530.0
Pancreatic cancer	112	238	471.1
Lung cancer	221	1,248	177.1

*Notes*: Table is based on Table 1 from Brower (2005) (28). US burden of disease (BoD) Data were derived from the WHO Global Burden of Disease (GBD) report from 2004 (29) (2010 GBD country data will not be released until September 2013). US NIH funding data were derived from NIH Research Portfolio Online Reporting Tools (RePORT) (30). DALYs=disability-adjusted life years.

Looking at research investments is only one way of measuring what R&D is being undertaken. Other R&D indicators can also be reviewed, such as the number of research articles or ongoing clinical trials (6, 19, 23, 31–34). By doing so, Nwaka et al., with regard to health R&D in Africa, recently showed that ‘diseases disproportionately affecting Africa are under-prioritised’ (31). Table 3 is based on some of their results and makes clear for the five diseases with the highest burden from Table 1 that the variations in numbers of African publications and African clinical trials roughly correspond to the variations in global R&D funding. In another example, Dear et al. show that variations in the R&D that is conducted for different cancers exist in other countries too, by demonstrating that in Australia ‘four of the five cancers that result in the greatest burden of disease had relatively few clinical trials’ (33).

**Table 3:** Distributions of research articles and clinical trial research across five neglected diseases in Africa

	Number of articles with at least one African author/million African DALY	Number of trials recruiting in Africa/million African DALY
Diarrhoeal diseases	9.1	0.2
Lower respiratory infections and meningitis	10.6	0.5
Malaria	59.7	6.6
HIV	53.6	4.5
Tuberculosis	82.8	4.1

*Notes*: To demonstrate how the distribution of R&D can be measured using different R&D indicators, numbers of African research articles and African clinical trials are related to African burden of disease for the five diseases with the highest burden of disease in Table 1. Numbers were calculated from Nwaka et al. (2010) (31). DALYs=disability-adjusted life years.

## Causes

The problem is, then, that the health R&D that is undertaken globally is not ‘needs-driven’. The global landscape of health R&D shows gaps; there are neglected populations and products. Besides the discrete distinction between neglect and non-neglect, there are marked variations in the amount of R&D that is conducted for different health problems. Finally, the R&D that is undertaken for a particular health problem does not always match the knowledge or product development that is most needed for that problem. What has caused this mismatch between the health R&D that is needed and that which is undertaken?

A rational approach to establishing and funding a global agenda for health R&D is illustrated in Box 2. In reality, there are problems with every step of this approach, together forming the reasons that the mismatch exists.

First, there is no system to comprehensively, systematically, and periodically map what health R&D is needed globally (step 1) (35). Health R&D needs, as detailed in Box 1, are determined by the burdens of existing health problems, by the need for new knowledge and products, and by the R&D that is already being undertaken. Although substantial progress has been made in evaluating the burdens of existing health problems (25, 29), the need for new knowledge and products is only assessed on an *ad-hoc* basis and for a selected number of diseases (12, 36–38). Our knowledge of what health R&D is being conducted, where it is being conducted, by whom and how, is also very limited (19, 27). Moreover, there is currently no accepted approach for comparing health R&D needs across different health problems (35).

Second, although health R&D priorities are regularly established for specific diseases and countries, there is currently no system to facilitate the prioritisation of all health R&D needs and the formulation of ‘best buys’ in health R&D globally (step 2) (39, 40).

Finally, there are problems with realising a coordinated response to established global priorities for health R&D (steps 3 and 4). The current global health R&D system relies strongly on market incentives. About 60% of all health R&D funding comes from the for-profit private sector (6). However, when market incentives drive innovation, R&D that is profitable will be preferred, with the neglect of populations and products that are not profitable as a result (10, 11). Market incentives even drive the development of products that may be profitable but that offer little or no additional therapeutic value (‘me-too’ drugs) (10). Furthermore, few measures exist to ensure that products are affordable, which is an ever-present challenge for universal access to medicines when one considers that the great majority of the global burden of disease is carried by populations in developing countries (25, 29). Finally, a lack of open innovation is inherent to a competitive, privatised system and constitutes an impediment to the efficiency and ethicality of the R&D system (10). This was recently demonstrated by the reluctance of pharmaceutical companies, with one exception (GlaxoSmithKline), to join the AllTrials campaign (an initiative that calls for all clinical trials to be registered and all trial results to be reported) (41).

**Box 2. A rational approach for establishing and funding a global health R&D agenda**

A rational approach for establishing and funding a global health R&D agenda consists of four steps (7, 11, 39): 
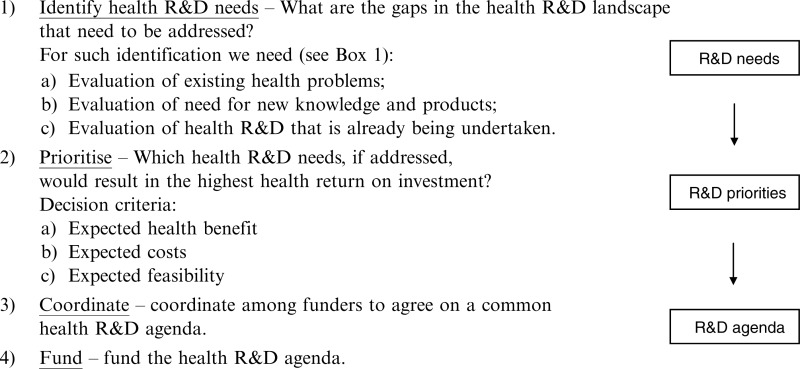

Public and philanthropic donors are responsible for the remaining 40% of all health R&D funding (6). In the area of neglected disease R&D, where more than 80% of R&D funding is allocated by these funders, the G-FINDER reports consistently show under-funding of priority areas of R&D and high-burden diseases (11, 12). How can it be that such gaps remain when public and philanthropic funders distribute the majority of funding?

One important reason is that there is not yet an accepted system of accountability for global health R&D needs. It is becoming increasingly recognised that the outputs of health R&D should be viewed as global public goods, meaning that all knowledge and products resulting from health R&D should be adapted and accessible to a global population of end users and that funding health R&D should be a globally shared burden (42). Yet, currently, there is no global governance arrangement that makes explicit the shared accountability that such views imply. In the absence of a concrete shared vision of accountability, the Bamako call to action on research for health suggests that in the current system all funders of health R&D are jointly responsible to ‘better align, coordinate, and harmonise the global health research architecture’ (43). However, in practice there are problems with regard to the degree to which these funders are accountable for global health R&D needs. The public and philanthropic health R&D funding landscape is diverse and includes national public funders of health R&D (such as health ministries or government research organisations), distributors of official development assistance (ODA) (such as government development or foreign affairs ministries), multilateral funding agencies, and philanthropic funders of health R&D (7, 12). National public funders of health R&D have often been established under national laws, have nationally focused remits, and are accountable to the parliament of the country they are based in. Hence, it is questionable whether they can be expected to fund health R&D that is globally relevant, but not of relevance to the country they are based in. Distributors of ODA do often have a global focus, but their contributions to overall global health R&D are relatively small as compared to funding by national public funders of health R&D (neglected disease R&D funding in the United States from 2000 to 2010, was funded predominantly by the NIH (87%) and much less so by the US Agency for International Development (6%) and the US Department of Defense (6%) (12, 44, 45)). The same is true for multilaterals (11, 12). In addition, multilaterals are often dependent on earmarked funding (46), and several multilaterals have remits that are limited to a specific set of diseases. Philanthropic funders of health R&D may also have a global focus, but given that they are privately funded, their accountability for global health R&D needs is, at best, uncertain (21). Tensions between global and national level priorities that arise because of the increasingly globalised nature of R&D, while most research funding is provided at a national level, are not unique to health (47).

Another important reason for the persistent nature of gaps in the global health R&D landscape is the lack of coordination by public and philanthropic funders on health R&D priorities. Given the fragmented funding landscape, enhanced coordination between funders on shared R&D priorities is greatly needed. However, such coordination currently only occurs selectively in particular areas (7, 40). There is no global ‘forum’ where funders comprehensively and periodically discuss priority health R&D needs and how to address those needs in a coordinated manner (40).

Finally, R&D funding allocation decisions by public and philanthropic funders, whether they have a national or a global remit, may be influenced by factors other than the need for health R&D (11, 20, 28, 48–51). Such factors include: the testimonials of patient advocacy groups or organisations with disease-specific mandates and advocacy and fundraising activities – ‘the squeaky wheel gets the grease’, as Brower suggests (11, 16, 28, 48); the presence of policy frameworks and funding mechanisms that prioritise specific diseases (11, 20); preferences of researchers (with most funders a large part of the research that is funded is investigator-initiated and some do not prioritise research areas at all) (28, 48, 49), to which the existence of trusted R&D groups, the institutionalisation of research topics, the attractiveness of research results, and the potential for publication contribute (11, 49, 52); the national values, interests, and political dynamics of the country in which the funder is based (20, 48, 50, 51); global values and political dynamics (20); community and media attention (28, 48, 49); and funder perceptions, preferences, and accountabilities (11, 48, 50). Given these diverse influences, there is a strong need for transparency from public and philanthropic health R&D funders on precisely what health R&D they fund and what their decision mechanisms are for funding allocation (21, 40). Funders themselves recognise the need for such transparency, as becomes clear from a recent joint statement from several large health R&D funders on the importance of sharing research data (53). Unfortunately, individual funders that provide publicly accessible statistics on past funding for different health and research categories are still an exception rather than a rule, and funders continue to apply a kaleidoscope of different research classification systems, making aggregate analysis of what funders fund exceedingly problematic (27).

## Solutions

The mismatch between the health R&D that is needed and the R&D that is undertaken has proven persistent over the past decades. Yet, solutions to this problem are available. The Consultative Expert Working Group on Research and Development: Financing and Coordination (CEWG) (7), an expert working group established by the World Health Assembly in 2010, released an extensive report in 2012 which provides recommendations for how to systematically identify global health R&D priorities and ensure that these are addressed in a coordinated manner. The starting point for realising this is described to be the establishment of a Global Observatory on Health R&D (6, 7, 19).

The mission of an Observatory is envisioned to include the mapping of health R&D needs, with the goal of establishing clarity on R&D priorities (‘best buys’), and the bringing together of health R&D funders to facilitate coordinated action on a shared R&D agenda. If these goals are to be reached, lessons would have to be learned from the shortfalls of the Global Forum for Health Research, an organisation with a similar mandate which was recently discontinued. Two lessons are of particular importance to the challenges that an Observatory could be faced with. First, although the Global Forum established a process for continuous monitoring of global investments in health R&D (44), it never succeeded in conducting a comprehensive mapping of the needs for new knowledge and products. Arguably, this is the most important step of any priority-setting process for health R&D and would need to be a focus of an Observatory (35, 39). Second, the most effective way to ensure that ‘best buys’ in health R&D are indeed funded would be to link an Observatory to a pooled funding mechanism, akin to the Global Health Research Fund once suggested by the Commission on Macroeconomics and Health (54). Such a fund could disperse funding to public, private, or public–private partnership research entities in areas of identified priority health R&D need (7). Should this prove unfeasible, then an alternative would be to bring together funders of health R&D to galvanise coordinated action on a shared R&D agenda (4, 7, 40). The Global Forum was established precisely to be a forum for such discussion, but never succeeded in actually bringing funders together to discuss ‘best buys’ in health R&D. To prevent a similar course of events with an Observatory, it will be essential to generate broad support for this new platform and to work together with key funders of health R&D in giving rise to the final shape and form of an Observatory (55). One way to do this would be to learn or even build from existing models of funder collaboration that have proven to be successful, such as the European and Developing Countries Clinical Trials Partnership (56) and ESSENCE on Health Research (Enhancing Support for Strengthening the Effectiveness of National Capacity Efforts) (57).

Strengthening national health research systems, in particular in those countries with the largest burden of disease, was already noted as being of particular importance to correcting the 10/90-gap in 1990 by the Commission on Health Research for Development (4). Their report lead to the establishment of the Council on Health Research for Development (COHRED) in 1993, an organisation whose mission is to ‘improve health, equity, and development by supporting countries to develop strong research and innovation systems’ (58). Yet, two decades later, despite significant efforts to improve countries’ health research systems, by COHRED (59) and others (60), this still constitutes a challenge of pressing priority (60). An envisaged additional advantage of an Observatory would be that it could provide an impetus for national health research system strengthening. It could do so by stimulating the development of good practices and standards in health research, by providing support for building capacity for health R&D in developing countries, by producing analyses to inform national R&D portfolio management, and by creating a platform to convene stakeholders (6, 55).

The pharmaceutical industry has developed more expertise with technologies for the conversion of basic scientific discoveries into new therapies than the public sector and the involvement of the for-profit private sector is thus of major importance in creating solutions to the mismatch (36). Many different approaches for engaging the for-profit private sector in targeting unprofitable R&D and for delinking the price of health R&D from its cost have been proposed and tested in recent years (10). Examples are product development partnerships, which have proven particularly effective for developing new products for neglected diseases (10, 45); other public–private partnerships, such as those recently announced by both the European Union and the United States that will aim to develop new antibiotics in the face of increasing antibiotic resistance (61, 62); economic incentives established through legislation, which have shown to be effective for stimulating R&D for paediatric medicines and orphan drugs (although only in part, with both orphan drugs and paediatric medicines concerns have been raised about using economic incentives, since the R&D that is stimulated through such measures remains driven by market incentives rather than by need) (14, 16, 63, 64); and different kinds of prizes and grants to companies, which are considered to be particularly effective for stimulating health R&D of relevance to developing countries (7).

Besides improving the prioritisation of health R&D needs, facilitating the coordination of public and philanthropic funders, strengthening national health research systems, and engaging the for-profit private sector, it will be necessary to increase access to research results and to improve research collaboration (through open approaches to R&D, equitable licensing, and patent pools) (7).

Finally, there is a need to gather these different measures under the umbrella of a concerted mechanism through the establishment of a global framework or convention on health R&D (7, 10) (the World Health Organization (WHO) has the option to create legally binding conventions on the basis of a two-thirds majority vote of its Member States, but has only done so once (65)). A framework or convention would provide the global governance framework to secure the nature of health R&D as a global public good, making explicit the globally shared responsibilities for addressing global health R&D needs and thus raising the financial resources needed to realise such sizeable changes to the global health R&D system (7, 42, 66). Notably, such funds would allow for the realisation of a pooled funding mechanism linked to an Observatory, providing an effective, coordinated, and sustainable source of funding for identified health R&D priorities (7, 67). The establishment of a framework or convention has been a much discussed topic in recent years (7, 10). Because countries would be expected to contribute financially based on their level of development (7, 42), while the R&D output would mainly benefit populations in developing countries, it has been a much contested proposal on which nations have stood divided. At the most recent World Health Assembly of May 2013, discussion on a framework or convention was postponed until 2016 (68, 69). This is a regrettable outcome after more than two decades of negotiations and reports by several expert working groups, who have all made sensible and rational suggestions to improve the world's health R&D system, but have been met with little action (4, 7, 70–73).

Although discussion on a framework or convention was postponed, the establishment of a Global Observatory on Health R&D was enacted at the most recent World Health Assembly (68, 69). Furthermore, WHO was requested to review possibilities for coordinating and financing global health R&D priorities (35, 67, 69) and to facilitate the implementation of several health R&D demonstration projects to address identified gaps that disproportionately affect developing countries (69, 74). These plans alone are not enough to address the substantive mismatch between the health R&D that is needed and that which is undertaken. Still, they constitute an important step forward and, looking ahead to the World Health Assembly in 2016, present an opportunity for demonstrating the value of more far-reaching changes to the global governance framework for health R&D. It is important that WHO takes immediate action to demonstrate that value, in particular through coordinating the selection and implementation of the health R&D demonstration projects.

## Footnotes

1 R&D is defined by the Organisation for Economic Co-operation and Development (OECD) as: ‘Research and experimental development comprise creative work undertaken on a systematic base in order to increase the stock of knowledge, including knowledge about man, culture, and society, and the use of this knowledge to devise new applications’ (1). R&D is generally subdivided into basic research, applied research, and experimental development. Health R&D includes fields such as epidemiology, health services and health systems research, and health-related social research (1–3).
